# Role of miR-182 in response to oxidative stress in the cell fate of human fallopian tube epithelial cells

**DOI:** 10.18632/oncotarget.5493

**Published:** 2015-10-12

**Authors:** Yugang Liu, Wenan Qiang, Xiaofei Xu, Ruifen Dong, Alison M. Karst, Zhaojian Liu, Beihua Kong, Ronny I. Drapkin, Jian-Jun Wei

**Affiliations:** ^1^ Department of Pathology, Northwestern University Feinberg School of Medicine, Chicago, IL, USA; ^2^ Department of Obstetrics and Gynecology, Northwestern University Feinberg School of Medicine, Chicago, IL, USA; ^3^ Robert H. Lurie Comprehensive Cancer Center, Northwestern University Feinberg School of Medicine, Chicago, IL, USA; ^4^ The Division of Medical Oncology, Dana Farber Cancer Institute, Harvard Medical School, Boston, MA, USA; ^5^ Department of Obstetrics and Gynecology, Qilu Hospital, Shandong University, Jinan, Shandong, China

**Keywords:** fallopian tube secretory cells, ROS-induced miRNA (ROSmiR), p53, senescence bypass, tumorigenesis

## Abstract

High grade serous ovarian carcinoma (HGSC) is a DNA instable tumor and its precursor is commonly found originating from the fimbriated end of the fallopian tube secretory epithelial (FTSE) cells. The local stresses via ovulation and related inflammation are risks for HGSC. In this study, we examined the cellular and molecular responses of FTSE cells to stress. We found that excess intracellular reactive oxygen species (ROS) in normal FTSE cells upregulated a subset of microRNA expression (defined as ROSmiRs). Most ROSmiRs' expression and function were influenced and regulated by p53, and together they drove the cells into stress-induced premature senescence (SIPS). However, ROS-induced miR-182 is regulated by β-catenin, not by p53. In normal FTSE cells, miR-182 overexpression triggers cellular senescence by p53-mediated upregulation of p21. Conversely, in cells with p53 mutations, miR-182 overexpression no longer enhances p21 but functions as an “Onco-miR”. p53 dysfunction is a prerequisite for miR-182-mediated tumorigenesis. In addition, we found that human follicular fluid could significantly induce intracellular ROS in normal FTSE cells. These findings suggest that ROS and p53 mutations may trigger a series of events, beginning with overexpressing miR-182 by ROS and β-catenin, impairing the DNA damage response, promoting DNA instability, bypassing senescence and eventually leading to DNA instable tumors in FTSE cells.

## INTRODUCTION

Despite the significant efforts of basic and clinical research, the survival rate of women with ovarian carcinoma has not changed significantly in the past fifty years [1]. High grade serous ovarian carcinoma (HGSC) is the most common type and is a deadly form of ovarian cancer and early diagnosis remains a challenge. HGSC encompasses DNA-instable tumors with frequent *BRCA1/2* [2] and *p53* mutations [3], wherein *p53* mutation alone is not sufficient to trigger a sequence of neoplasia [4]. A recent mouse model by combining inactivation of *brca/p53/pten* produced tumors mimicking human HGSC [5] indicates these tumor suppressor genes are critical in the development of HGSC.

Recent studies suggest that some miRNAs are sensitive to oxidative stress (OS), and that ROS exposure can induce the expression of specific microRNAs [6, 7]. These miRNAs react to stresses through coordination of the target gene regulation. Interestingly, most stress-induced miRNAs are mediated by *p53* [8]. It can be speculated that the stress-induced miRNA expression and *p53* function may play a central role in determining the cell fate and may trigger sequential and as of yet, not fully characterized pathways, thus increasing the risk for HGSC transformation. Fallopian tube secretory epithelial (FTSE), but not ciliate (FTCE) cells are the cell origin of HGSC [9]. HGSC precursor lesions, known as serous tubal intraepithelial carcinoma (STIC) [10, 11], exist in the distal (fimbriated) ends of the fallopian tubes but are rarely seen elsewhere. While the mechanisms for why FTSE at fimbriae are the targets of HGSC remain largely unknown, as local microenvironmental stress induced by ovulation is a risk factor for ovarian cancer [12]. Monthly ovulation may produce trauma-induced inflammation and unbalanced ROS for local OS [13]. Cellular response to microenvironmental stresses is intricately regulated by a complex network of molecules. While stress-induced premature cellular senescence (SIPS) has been considered as a protective mechanism against tumorigenesis as the accumulation of cells undergoing SIPS may contribute to double strand DNA breaks, mitochondrial OS injury, chronic inflammation, abnormal proliferation and cellular transformation [14]. How normal and defected FTSE cells respond to OS should be investigated.

To investigate how FTSE cells react to stress, in this study, we examined ROS-induced miRNA (we defined as ROSmiRs) dysregulation and their molecular regulation mechanism; miRNA functions in response to OS in the presence and absence of *p53*; and the major stress mediator ROS, which is highly induced by follicular fluid and the inflammatory cytokines. We found that FTSE cells were vulnerable to local stress and the ROSmiRs play a critical role in normal and abnormal FTSE cells. Disrupting the balance between stress and miRNA expression, especially *miR-182*, results in the impaired DNA damage response, senescence bypass, risk for tumor transformation and eventual development of DNA instable HGSC.

## RESULTS

### ROS- induced miRNA (ROSmiR) and mRNA expression in FTE cells

**Figure 1:**
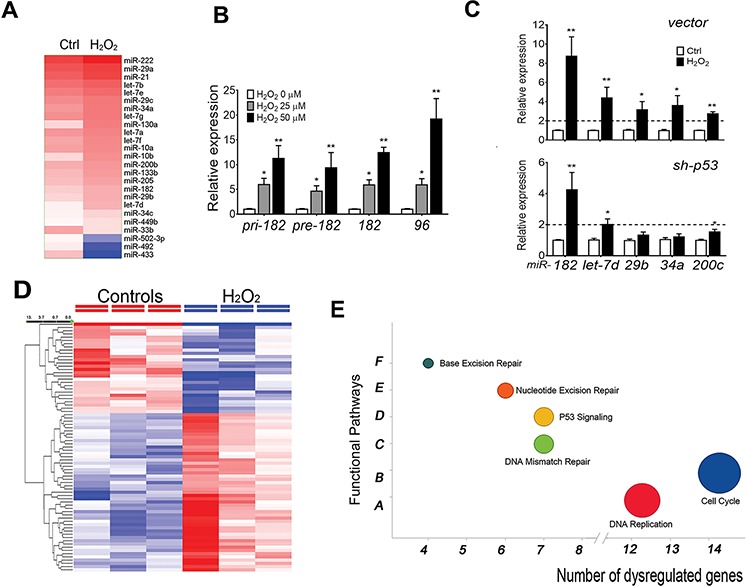
ROS-induced miRNA (ROSmiRs) and mRNA expression in FTE cells **A.** Dendrogram illustrated ROS-induced miRNAs (ROSmiRs, >1.5 fold dysregulation) in primary FTE cells (*n* = 3) treated with DMSO (Ctrl) and ROS (H_2_O_2_ 50 μM). **B.** ROSmiR expression including pri-, pre- and mature *miR-182* and its family member *miR-96* when primary FTE cells were treated with different concentrations of H_2_O_2_ (0: open box, 25 μM: gray box; 50 μM: dark box). **C.** The selected ROSmiR expression in primary FTE cells by blocking *p53* (lower panel) expression (*sh-p53*) and vector control (upper panel) followed by H_2_O_2_ (100 μM) for 24 hours. **D.** Dendrogram illustrated top dysregulated genes (>1.5 fold) in primary FTE cells (*n* = 3) treated with DMSO (control) and ROS (H_2_O_2_ 50 μM). **E.** Pathway analysis of ROS-induced gene expression of primary FTE cells in cell function pathways. *p < 0.05, **p < 0.01, ***p < 0.001.

Many studies used H_2_O_2_ to mimic the function of ROS/superoxide in different type of cells. To explore ROSmiRs expression in FTE cells, a global miRNA profiling analysis in cultured primary FTE cells was examined. We found a subset of miRNAs that were significantly dysregulated in FTE cells after H_2_O_2_ exposure including *let-7s, miR-34s,* and *miR-200s* and *miR-182* family members (Figure 1A, Suppl Table 1, Suppl Figure 1A). Moreover, upregulation of three different forms of *miR-182* (pre-, pri- and mature) and *miR-182* family members (*miR-96* and *miR-183*) suggests ROS-induced *miR-182* is at transcriptional level and dose dependent in primary FTE (Figure 1B) and immortalized FTSE cell lines (Suppl Figure 1B).

Many ROSmiRs (*let-7s, miR-34s* and *miR-200s)* seemed to be *p53* dependent [8]. To investigate whether ROS induced miRNA expression was *p53* dependent, we first examined ROSmiR expression in FTSE cell lines. FTE237 was immortalized by *sh-p53* (Suppl Table 2) and absent of *p53* expression, while FTE194 had moderate TP53 expression. When both cells were treated by 100 μM H_2_O_2_ for 24 hours, *miR-182* upregulation was noted (data not shown). To determine the role of *p53* in ROSmiR expression, the primary cultured FTE cells were prepared in *ex vivo* culture, in which, *p53* was blocked by *sh-p53*. Similar to FTSE cell lines, cells were treated with 50 μM H_2_O_2_ and ROSmiR expression were examined by real-time RT-PCR (Figure 1C). Both *miR-182* and *miR-200c* were inducible when *p53* was present, but only *miR-182* was significantly induced by H_2_O_2_ when *p53* was blocked by *sh-p53*. We found among most ROSmiRs, *miR-182* was highly induced by ROS in cells with and without *p53* expression.

ROS resulted in significant gene alterations in primary culture FTE cells. Gene profiling analysis revealed a total of 88 genes were upregulated and 134 genes were downregulated by ROS (*p* < 0.05, Figure 1D, Suppl Table 3A and 3B). Further analysis showed that ROS induced gene dysregulation involved mainly in cell cycle, DNA replication, *p53* signaling pathway (Figure 1E).

Taken together, ROS induces a subset of miRNAs in FTE cells and most of them are mediated by *p53* expression, but *miR-182* is an exception.

### ROS- induced premature cellular senescence through ROSmiRs

**Figure 2:**
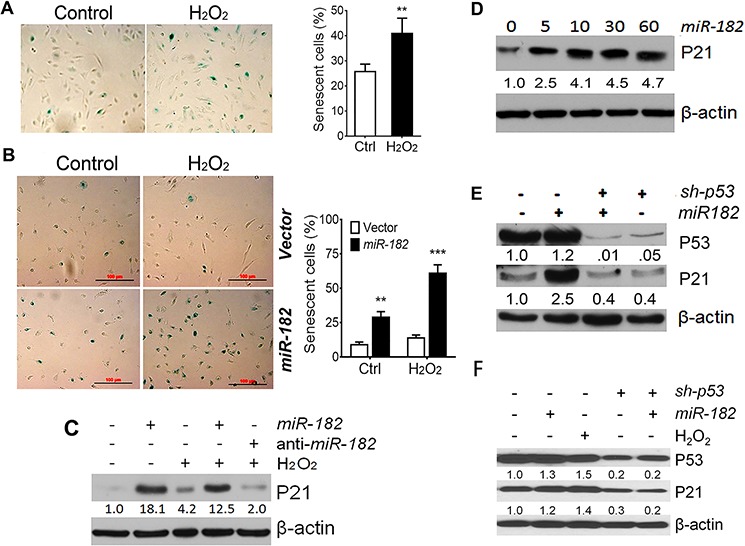
ROS-induced premature cellular senescence is mediated by ROSmiRs and cell cycle genes **A.** ROS (H_2_O_2_ 50 μM)-induced premature cellular senescence in primary FTE cells was detected by SA-β-gal staining (left) and counted senescent rate (percentage) in control (Ctrl, open box) and H_2_O_2_. **B.** The percentage of senescent cells was counted in FTE194 cells with (dark box) and without (open box) *miR-182* overexpression. Three randomly selected fields (1 × 1 mm^2^) of images were captured to count the senescence rate (%). **p < 0.01, ***p < 0.001. **C.** P21 expression were examined by western blot in FTE194 cells by controlling *miR-182* expression and treated with H_2_O_2_ (100 μM). **D.** P21 expression in FTE194 cells by administrations of different doses of miR-182 mimic (0–60 nM). **E and F.** P21 and P53 expression in uterine smooth muscle cell line, DD-HLM (E) and FTE194 (F) cells by controlling *miR-182* expression, blocking *p53* expression by *sh-p53* and treating with H_2_O_2_. Protein band intensity was measured by density photometry and is listed below. β-actin was used as a protein loading control.

ROS can be double-edged swords in reproductive organs depending on their level and intensity in cells [13]. The effects of oxidative stress on female reproduction and related diseases involve many different pathophysiologic mechanisms [13]. The major effects of ROS on cellular toxicity include DNA stress, SIPS/cell cycle arrest, and cell death. To evaluate the cellular response of ROS in FTE cells, we simply exposed H_2_O_2_ in different dosages in primary FTE and immortalized FTSE cells. 50 μM and higher H_2_O_2_ triggered SIPS of FTE cells (Figure 2A). This is consistent with previous reports on many ROSmiR (*miR-34s, miR-200s* and *let-7s*) in different cell types [15]. In this study, we overexpressed *miR-182* in FTSE cell lines and found *miR-182* overexpression can trigger significant cellular senescence in primary FTE and immortalized FTSE cells (Figure 2B, Suppl Figure 2A and 2B).

Among the top dysregulated ROSmiRs in FTE cells (Figure 1A), *miR-34s, miR-200s* and *let-7s* have been reported to regulate cell cycles and promote SIPS. We recently reported that overexpression of *miR-182* [16] and *miR-200a* [17] can trigger significant cellular senescence in uterine leiomyoma. These findings prompted us to test whether ROS-induced SIPS is through ROSmiRs. We overexpressed *miR-182* in FTSE cell lines and examined cellular senescence and several cell cycle negative regulators in primary and immortalized FTE cells. *p21* was one of the major genes influenced by *miR-182*. When introducing *miR-182* overexpression in FTE194, we noted not only the triggering of significant cellular senescence (Suppl Figure 2A) but also upregulated P21 expression with or without ROS exposure (Figure 2C, Suppl Figure 2C). Moreover, *miR-182* mediated P21 upregulation was dose dependent (Figure 2D). Furthermore, the level of P21 expression by *miR-182* was much higher than simply ROS exposure and anti-*miR-182* treatment was able to block P21 expression (Figure 2C, Suppl Figure 2C). Interestingly, *miR-182* mediated P21 upregulation was not observed in cell line FTE237, which was immortalized by sh-p53, and no detectable *p53* expression was noted (Suppl Figure 2D) [18]. To assess whether *miR-182* mediated P21 upregulation requires P53, *p53* expression was inhibited by lentiviral *sh-p53* in leiomyoma cell line DD-HLM (Figure 2E) and FTE194 (Figure 2F) and then overexpressed *miR-182*. We noted that only blocking *p53* expression resulted in abolishing *miR-182* mediated P21 upregulation in leiomyoma cells (Figure 2E) and FTSE cells (Figure 2F). Findings strongly suggest that *miR-182* triggers SIPS by upregulation of *p21* and this process requires functional *p53*.

### ROS or stress-induced miR-182 is through β-catenin when P53 is inactivated

**Figure 3:**
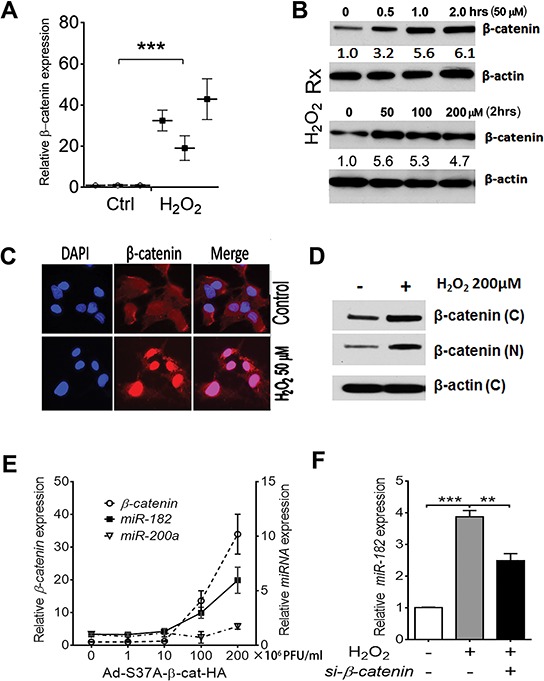
ROS or stress-induced *miR-182* expression is regulated by β-catenin **A.** *β-catenin* mRNA expression in primary FTE cells (*n* = 3) treated by DMSO (ctrl) and H_2_O_2_ detected by real time RT-PCR. **B.** Time (0–2 hours) and dose (0–200 μM) effect of H_2_O_2_ on β-catenin protein expression detected by western blot in the FTE194 cell line. **C.** Immunofluorescent image analysis of β-catenin expression (counterstained with DAPI, blue color) and cellular location in FTE194 cells with and without H_2_O_2_ exposure. **D.** Western blot analysis of β-catenin expression in FTE194 cells extracted from nuclear (N) and cytoplasmic (C) fraction with and without H_2_O_2_ exposure. β-actin was used as a protein loading control. **E and F.** To determine whether *β-catenin* specifically regulated *miR-182* expression, β-catenin expression was controlled by different doses of *β-catenin* in the quantitative adenovirus infection by *β-catenin* construct Ad-S37A-β-cat-HA (×10^6^ PFU/ml) in FTE194 cells. *β-catenin* (hollow dot), *miR-182* (dark square), and *miR-200a* (triangle dot) expression was detected by real time RT-PCR (E). *MiR-182* expression was further examined when *β-catenin* expression was inhibited by si-RNA (F). *p < 0.05, **p < 0.01, ***p < 0.001.

As illustrated above, ROS significantly induces *miR-182* overexpression (Figure 1). *MiR-182* overexpression further triggers SIPS through upregulation of *p21* in the presence of *p53* (Figure 2). Other ROSmiRs, including *miR-34s, miR-200s* and *let-7s*, also contribute to ROS-induced senescence and *p21* upregulation [19–21]. Given that most of the ROSmiRs are directly or indirectly regulated by *p53* [8] (Figure 1E), and *p53* is frequently mutated during the early and later stages of high-grade serous carcinoma, the role of *p53* independent ROSmiR, such as *miR-182* expression and its function in FTE cells, seems to be pivotal when *p53* is partly or completely inactivated. To explore what molecular mechanisms are involved with ROS-induced *miR-182* expression in FTE cells, we examined several candidate genes that respond to ROS and potentially regulate *miR-182* expression. Initial screening showed that β-catenin was significantly upregulated by ROS (Suppl Figure 3A) and/or h-FF (Suppl Figure 1C). Further study showed that β-catenin is constantly upregulated when administering ROS in *ex vivo* culture of primary FTE cells (Figure 3A). ROS upregulates *β-catenin* expression can also be repeated in FTSE cell line (Figure 3B, Suppl Figure 3B). ROS upregulating β-catenin could be detected at 30 min after ROS exposure and reach to peak at 2 hours (Figure 3B), and seemed to be dose dependent (Suppl Figure 3B). Immunofluorescent staining revealed that ROS-induced β-catenin had significant cytoplasmic to nucleus translocation (Figure 3C) and this could be further confirmed by western blot analysis (Figure 3D).

To evaluate how β-catenin regulates *miR-182* expression in FTE cells, *β-catenin* was overexpressed by introducing the adenovirus *β-catenin* construct Ad-S37A-β-cat-HA in FTSE cell lines, and *miR-182* expression was examined. We found β-catenin upregulated *miR-182* expression, and it was dose dependent (Figure 3E, Suppl Figure 3). When repeating the same experiment to examine other ROSmiR (*miR-200*a) expression by *β-catenin*, no significant change was found (Figure 3E). When blocking *β-catenin* expression by si-RNA, ROS-induced *miR-182* expression was significantly reduced (Figure 3F). Findings suggest that ROS-induced *miR-182* expression is mostly through ROS-induced *β-catenin* expression and its nuclear translocation.

### Upregulation of miR-182 enhances senescence bypass in FTSE cells when exposed to ROS or DNA stress

**Figure 4:**
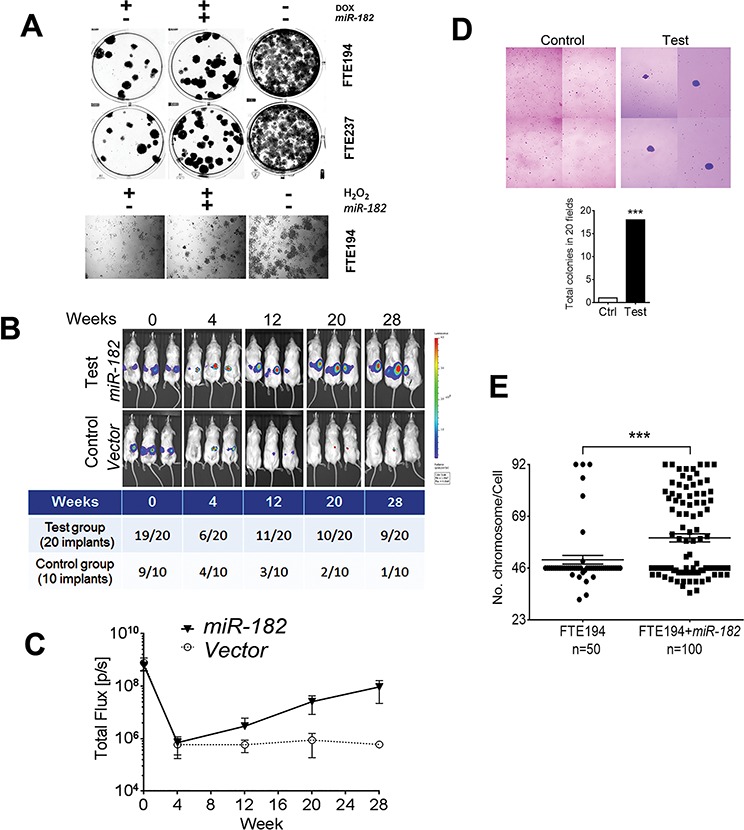
*miR-182* enhances senescence bypass in FTSE cells under DNA stress and impaired *p53* **A.** Senescence bypass was evaluated by colony formation assay in FTE194 and FTE237 cell lines with and without *miR-182* overexpression after doxorubicin (DOX, 0.2 μg/ml) or H_2_O_2_ (100 μM) treatment. **B and C.** Senescence bypassed FTE194 cells with (test) and without (control) *miR-182* overexpression were stably transfected with luciferase and grafted into the intrabursa in nude mice (B). Cell growth was monitored by IVIS weakly, up to 180 days. The number of detectable xenografts (B) and cell growth rate (C) were measured and counted. **D.** Soft agar assays showed that chronic stress treatment in FTE194 and FTE237 cells with (test) and without (control) *miR-182* overexpression increased anchorage independent growth. **E.** FTE194 cells with and without *miR-182* overexpression were chronically treated with low dose of H_2_O_2_ exposure (10 μM) in each passage and up to 15 passages. Karyotypes were prepared and analyzed in FTE194 (*n* = 50) and FTE194 with *miR-182* overexpression (*n* = 100) and the aneuploidy was counted and summarized in dotplots (bottom). ***p < 0.001.

Expression of senescence-associated ROSmiR in FTSE cells requires p53 when exposed to ROS [8] (Figure 1). When p53 is intact, these ROSmiRs including *miR-182* function as tumor suppressors or cell cycle inhibitors by triggering SIPS (Figure 2). However, when p53 is lost, ROS upregulates *miR-182* through the *β-catenin* pathway, but *miR-182* mediated *p21* upregulation is aborted (Figure 2E, 2F). How do the FTSE cells react to stress when p53 is lost or mutated? To address this question, we established stable overexpression of *miR-182* in the immortalized FTSE cell lines. The same cell lines without *miR-182* overexpression were used as controls. All control and test cells were treated with Doxorubicin (DOX), a potent DNA damage reagent, and we found significant SIPS in all cell types (Suppl Figure 4A). However, in addition to higher senescent rate in cells with *miR-182* overexpression (Suppl Figure 4A), these cells also obtained a significantly high-rate of senescence bypass when treated with ROS or DOX (Figure 4A).

To further evaluate the cellular nature of the senescence bypassed FTSE cells with and without *miR-182* overexpression, FTE194 cells were stably overexpressed with luciferase and grafted into the intrabursa in nude mice (for detailed techniques, please refer to our recent publication [22]). We used IVIS to monitor the cell growth each week (Figure 4B). At the end of 180 days, cell growth was detectable in 45% (9/20) of FTSE cells with *miR-182* overexpression and only in 10% (1/10) of implants in the control group (Figure 4C). The data from FTE237 cells were similar (data not shown). Although no tumor formation was noted in all of the mice, long-lasting cell growth of FTSE with *miR-182* overexpression suggesting the role of promoting cell proliferation and independent growth by *miR-182* when *p53* is partly inactivated (Figure 4C).

One of the major functions for *miR-182* involves the DNA damage response [23]. To investigate the role of *miR-182* in DNA integrity and the chances of tumor transformation, we isolated the senescence bypass FTSE cells with and without *miR-182* overexpression and treated with lower doses (25–50 μM) of H_2_O_2_ in each passage and propagated up to 15 passages (to mimic chronic stress *in vitro*). These chronically stressed cells with *miR-182* overexpression can occasionally transformed *in vitro*, detected by soft agar (Figure 4D). DNA alterations were characterized by a significant increase of aneuploidy in FTSE cells with *miR-182* overexpression (Figure 4E, Suppl Figure 4B).

### Different reactions to ROS and DNA damage response between FTSE and FTCE cells

**Figure 5:**
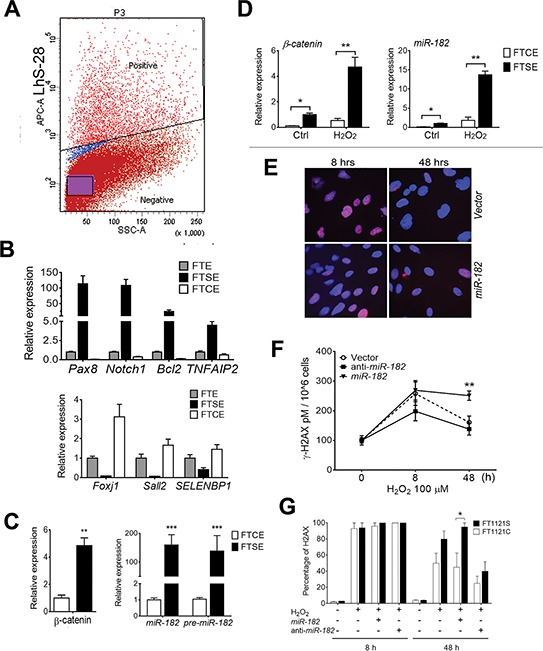
Differential reaction to ROS and DNA damage response between FTSE and FTCE cells **A.** FTSE and FTCE cells were sorted by flow cytometry BD FACSAria 5-Laser in *ex vivo* cultured primary FTE cells labeled by APC conjugated anti-LhS28. **B.** The unsorted FTE cells (gray box) and sorted FTSE (dark box) and FTCE (open box) cells were subjected to cell type specific biomarker analysis by real-time RT-PCR for secretory cells (top panel) and ciliated cells (bottom panel). **C and D.** *β-Catenin* and *miR-182* expression was detected in the sorted FTSE and FTCE cells with and without ROS treatment. **E and F.** DNA damage response in *ex vivo* culture of total FTE cells with or without *miR-182* transient transfection. Cells were treated with H_2_O_2_ (50 μM) and DNA damage was examined by immunofluorescent staining of γ-H2A.X at time points of 0, 8 and 48 hours. Total protein was extracted from cell pellets and added to 96 well plate coated with γ-H2AX antibody. Goat anti-Mouse IgM HRP Conjugate was used and results were taken by chemiluminescent readings using a Cytation 3 Cell Imaging Multi-Mode Reader in three time points for control (open dot), anti-*miR-182* (dark square) and *miR-182* mimic (triangle). Determination of γ-H2AX concentrations was done and shown as pM/106 cells. **G.** DNA damage response in *ex vivo* culture of the sorted FTSE (dark box) and FTCE (open box) cells with or without *miR-182* overexpression (by transient transfection 60 nM *miR-182* mimic) and anti-*miR-182* treatment. DNA damage was examined by immunofluorescent staining of γ-H2A.X at time points of 0, 8 and 48 hours. The positive cells with γ-H2A.X staining (> 10 dots/nucleus) were counted. *p < 0.05, **p < 0.01, ***p < 0.001.

Normal fallopian tube consists of two major cell types, secretory (FTSE) and ciliated (FTCE) surface epithelial cells. Compelling evidence supports that most high-grade serous carcinoma arise from FTSE cells [10, 11]. Recent study showed that FTSE cells seemed to have a delayed DNA damage response (DDR) in comparison to FTCE cells [18]. To further investigate whether these two cell types react differently to ROS, we examined the stress response in FTSE and FTCE cells. When *ex vivo* primary culture of FTE cells was established (Suppl Figure 5A), pure epithelial cells gated by anti-CD326 (EpCAM). FTE cells were then sorted with anti-LhS28-APC, a ciliate surface protein, by flow cytometry (Figure 5A). Overall, 15–30% of cells were FTCE cells. To further evaluate the sorting results, the sorted cells were subjected to RT-PCR analysis in the selected FTSE and FTCE cell markers. The gene expression patterns in the sorted cells indicated that the sorting was successful (Figure 5B). The cells were also evaluated by immunofluorescent stain of PAX8 (secretory marker) and LhS28 (ciliated marker) (Suppl Figure 5B). We found that the baseline β-catenin and *miR-182* family members were higher in FTSE than in FTCE cells (Figure 5C).

Next, the sorted FTSE and FTCE cells were exposed to different doses of ROS for 24 hrs. While exposed to 50 μM ROS, both cell types had elevated β-catenin, but FTSE cells had three to seven fold higher β-catenin than FTCE cells (Figure 5D). Consistent with β-catenin reaction, *miR-182* and its family members were also significantly higher in FTSE cells than in FTCE cells when exposed to ROS (Figure 5D). Findings suggest that FTSE and FTCE cells react differently to ROS and ROS induced genes.

*MiR-182* overexpression significantly inhibits DNA damage response through its negative regulation of DNA repair genes, *BRCA1* and *FOXO3a* [23, 24]. In this study, we first examined DNA damage repair dynamics in FTE194 cell lines. The cells with and without miR-182 overexpression treated with ROS and *anti-miR-182*, DNA damage repair were examined by immunofluorescent stain, H2AX at time points of 0, 8 and 48 hours. The levels of H2AX immunoreactivity were examined by fluorescent microscope (Figure 5E) and ELISA-based intensity measurement (Figure 5F). Apparently, miR-182 overexpression significantly slowed DNA repair after 48 hours of ROS exposure. Next, we examined DNA damage response (DDR) between FTSE and FTCE cells. The positive cells with H2AX stain were counted. We found FTSE cells had less than 10% of DNA repair, in contrast, FTCE had nearly 50% of DNA repair after 48 hours of ROS exposure. In the same experiment setting, transient transfection of *anti-miR-182* along with ROS exposure would significantly improve DDR to 55% of FTSE cells and 70% of FTCE cells at 48 hours, respectively (Figure 5G). Findings suggest that higher levels of β-catenin and *miR-182* expression in FTSE cells slow down DNA damage repair and this may result in increasing the risk of DNA alteration.

### Follicular fluid - induction of ROS in FTSE cells

**Figure 6:**
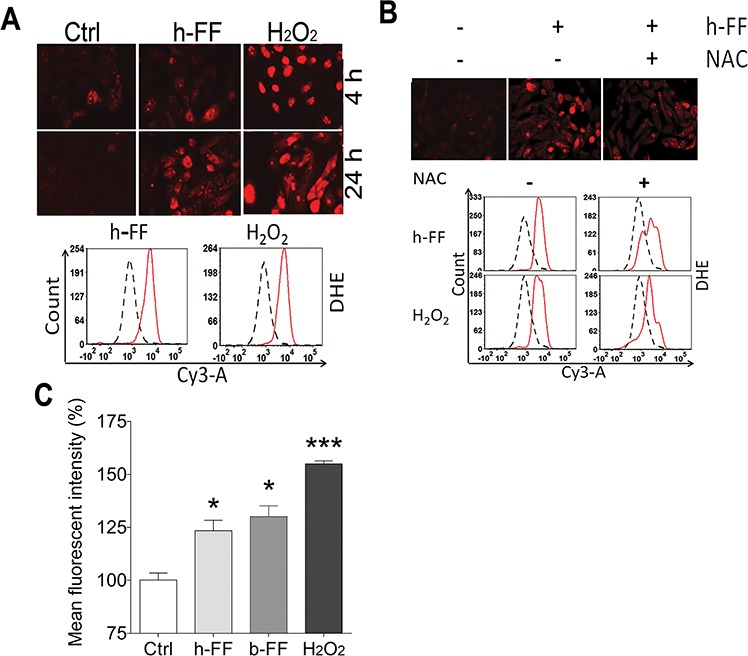
Follicular fluid for ROS induction in fallopian tube epithelial cells **A.** Human follicular fluid (h-FF, 50 μl/ml) induced ROS production in *ex vivo* culture of primary FTE cells at 4 and 24 hours. ROS intensity was detected by DHE (10 μM) stain and observed under a fluorescence microscope at excitation and emission wavelengths of 518/605 nm. H_2_O_2_ (50 μM) treatment was used as a positive control. To quantify ROS production, the fluorescent intensity was measured by flow cytometry. The plotting histogram showed the cell counts of the test (red solid line) and control (black dashed line) in cells treated with h-FF (left) and positive control of H_2_O_2_ (right). **B.** NAC (5 mM) was added 1 hour prior to h-FF or H_2_O_2_ treatment. The intensity of ROS was illustrated under the fluorescence microscope (upper) and plotting histogram (bottom). **C.** The histograms summarized the quantitative measurement of mean fluorescent intensity of ROS productions induced by h-FF and b-FF. Non-treated cells (open box) and H_2_O_2_ (50 μM) treated cells (dark-black box) were used as negative and positive controls. The data was generated in different cases. *p < 0.05, ***p < 0.001.

Monthly ovulation in women produces local stress and trauma on the ovarian surface. FTSE cells at the fimbriated end are directly exposed to human ovulatory follicular fluid (h-FF) and the local inflammatory environment [25]. It is well recognized that ovulation is a major stressor for the surrounding cells, causing trauma-induced inflammation and local oxidative stress [12]. To investigate how the FTE cells react to h-FF and inflammatory cytokines, we examine ROS production in primary and immortalized FTE cells. The primary FTE cells were exposed to 50 μl/ml h-FF for up to 24 hours, and a significant increase in ROS production was noted (Figure 6A). The finding was repeatable in at least three independent experiments from the different cases. To quantify ROS production, ROS intensity was measured and plotted by flow cytometry (Figure 6A). Likewise, similar findings were observed in immortalized FTSE cell lines (Suppl Figure 6A). To evaluate h-FF induced ROS in FTE cells, we treated h-FF with 56 and 96°C and similar results were obtained (data not shown). To determine whether cellular fluorescent signals induced by h-FF were from ROS, cells were treated with ROS inhibitor, NAC. As shown in Figure 6B, 5 mM NAC can mostly block h-FF induced ROS in cultured cells that are supportive of ROS production by h-FF. Consistent with h-FF, we observed that Bovine FF (b-FF) had a similar nature in FTSE cell ROS (Suppl Figure 6A).

The quantitative measurement of ROS production induced by h-FF, b-FF and controls were scored and summarized in Figure 6C (flow intensity) and Suppl Figure 6B (manually counted).

We also examined whether h-FF or b-FF has any effect on *miR-182* and *p53* expression in cultured FTE cells. Results shown that h-FF or b-FF in *ex vivo* culture FTE cells increased *miR-182* and *p53* expression (Suppl Figure 6C). Thus, h-FF can not only induce ROS production, but also increase the *miR-182* and *p53* expression in cultured FTE cells.

## DISCUSSION

The compelling evidence suggests that most HGSC arise from FTSE cells at the fimbriae [9]. In addition to the minority of patients with occasional genetic alterations [26], the causes of the disease remain largely unknown. Unbalanced high levels of ROS production can induce the expression of ROSmiRs (Figure 1). Since most ROSmiRs are either directly or indirectly regulated by *P53, p53* independent ROSmiRs should be critical in FTE cells when *p53* is mutant. ROS or DNA stresses can robotically upregulate *miR-182* [16, 27]. Unlike other ROSmiRs, ROS-induced *miR-182* does not require *p53* in some cell types [8, 28] as well as in normal FTSE cells observed in this study (Figure 1). In FTSE cells with normal *p53, miR-182* overexpression triggers SIPS by upregulation of *p21* (Figure 3). *P21* (*CDKN1A*), encoding a potent cyclin-dependent kinase inhibitor, is a regulator of cell cycle progression at G1 through inhibiting the activity of cyclin-CDK2 or -CDK4 complexes. *P21* is tightly controlled by *p53*, through which this protein mediates the *p53*-dependent cell cycle G1 phase arrest in response to a variety of stress stimuli, including ROS [29]. We have observed that administering ROS or simply overexpressing *miR-182* induces *p21* upregulation and triggers SIPS in FTSE cells (Figure 2). Our findings suggest that *miR-182* plays a central role in coordinating with *p53* to regulate *p21* for cellular senescence and to maintain DNA integrity in ROS-exposed cells.

In FTSE cells with *p53* mutations, *miR-182* overexpression no longer enhances *p21*, but may function as an “Onco-miR” through the repression of several tumor suppressor genes, including *BRCA1* [30] and *FOXO3a* [31], *FOXO1* [32, 33], *MITF1* [34], *MTSS1* [23], and *RECK* [35]. *MiR-182* is overexpressed in most HGSC [23, 36] and its overexpression is associated with aggressive tumor growth and the worst clinical outcome [23]. Therefore, ROS or DNA stress-induced *miR-182* and other ROSmiR overexpression requires *p53* for cell protection. We suggest that *p53* dysfunction is a prerequisite for *miR-182*-mediated tumorigenesis through negative regulation of DNA repair and cell cycle pathway [22, 23]. Our study for the first time, illustrates the dual effects of *miR-182* in FTSE cells and how these are closely related to the intactness of *p53* function.

To evaluate how the FTSE cells react to stress when *p53* is partly or completely lost or mutated, we established stable overexpression of *miR-182* in the immortalized FTSE cell lines. DOX, a potent DNA damage reagent, induces significant SIPS in all cell types. However, in addition to higher senescent rate in cells with *miR-182* overexpression, these cells also obtained a significantly high-rate of senescence bypass when treated with ROS or DOX (Figure 4). Although we would like to test the cell lines with wild type P53, this kind of cell line is currently not available. To further evaluate the cellular nature of the senescence bypassed FTSE cells with and without *miR-182* overexpression, FTE194 cells were stably overexpressed with luciferase and grafted into the intrabursa [22]. Although no tumor formation was noted in all of the mice by histologic evaluation, IVIS data showing long-lasting cell growth of FTSE with *miR-182* overexpression suggests the role of promoting cell proliferation and independent growth by *miR-182* when *p53* is partial inactivated (Figure 4). Current genetically engineered mouse models of ovarian cancer apply different combinations of MISRII-Cre, BRCA1/2, p53, Kras, PTEN, Dicer, Pax-8, et al [5, 37, 38]. One of the major functions for *miR-182* involves the DNA damage response [23]. The chronically stressed cells with *miR-182* overexpression can occasionally transformed *in vitro*, detected by soft agar. DNA alterations were characterized by a significant increase of aneuploidy in FTSE cells with *miR-182* overexpression (Figure 4).

**Figure 7:**
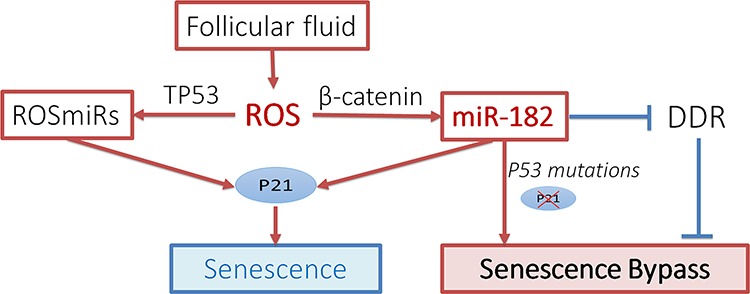
Proposed pathway for ROSmiR Sketch diagram illustrated the proposed pathways for ROS-induced miRNA (ROSmiRs) expression and cellular responses in fallopian tube secretory epithelial cells. DDR: DNA damage repair.

Current study also shows that FTSE and FTCE cells react differently to ROS-induced *β-catenin* and *miR-182* expression (Figure 5). This difference may in part explain the different repairing rate of DNA damage between the FTSE and FTCE cells identified in this study and the previous report [18]. This difference provides a strong clue for the potential functional role of *miR-182* in the early tumorigenesis of HGSC when *p53* is mutant. We demonstrated that in the presence of normal P53, stress-induced miR-182 overexpression mediated by β-catenin upregulates P21 and this will likely drive cells into SIPS. However, in cells with impaired or null p53, ROS induced miR-182 can increase the chance of senescence bypass (no-longer upregulating P21) and promote cell transformation or colony formation. Taken together, our current study suggests that the response to elevated ROS or DNA stress in FTSE cells may trigger a series of events, beginning with stress-induced *miR-182* expression and *p53* mutations, leading to impairments of the DNA damage response, DNA instability, bypassing senescence, thus increasing the risk of HGSC transformation (Figure 7) [4, 23].

The fimbriae are constantly exposed to environmental stresses (ovulation and pelvic inflammation). It is well recognized that ovulation is a major stressor for surrounding cells, causing trauma-induced inflammation and local oxidative stress [12]. The effects of oxidative stress on female reproduction and related diseases involve many different pathophysiologic mechanisms [13], however, the mechanisms behind ROS-mediated ovarian cancer development have not yet been investigated. h-FF is a complex body fluid and contains factors including steroid hormones, growth factors, pro-inflammatory cytokines and interleukins, prostanoids and proteolytic enzymes [7, 39]. Therefore, ovulation is considered to be comparable to an inflammatory reaction and to be the major risk factor for ovarian cancer [40]. Prolonged and repeated exposure to an inflammatory environment can lead to increased DNA adduct formation and gene mutations and risk for malignant transformation [41, 42].

However, ovarian cancer often manifests itself after risk factors such as ovulation have come to an end and supposedly protective hormones i.e. progesterone against estrogen have gone to rise is a puzzle at the crux of the ovarian cancer problem [25]. We propose that follicular fluid exposure leads to DNA double stranded breaks and induces high expression of *TP53*. Constant DNA stress triggers *TP53* mutations which is frequently seen in FTSE cells, defined as *p53* signature [43, 44]. *p53* signature does not present cellular malignancy features, but can be an early precursor lesion and may even take years (10–15 years as seen in most epithelial precursor lesion to malignant transformation) to harbor additional molecular changes and to develop occult STIC and invasive carcinoma. Therefore, understanding the link between the temporary induction of *TP53* and the related molecular chain reaction in response to h-FF exposure and the acquisition of mutations in *p53* in early precursor lesions will be the key for understanding the early tumorigenesis of HGSC [25]. In this study, we found h-FF can significantly induce a high level of ROS production and increase *miR-182* and *p53* expression in FTSE cells (Figure 6). Further study of the role of h-FF and its components in early precursor lesion of ovarian cancer are warranted. Future studies will be focused on developing the screening tests for miR-182 and mutant p53 expression in FTE cells for risk and non-risk populations and establishing a mouse model with overexpression of miR-182 and mutant p53 in FTE cells for ovarian cancer development.

## MATERIALS AND METHODS

### Human fallopian tube tissues and cell lines

Human fallopian tube tissues were collected from premenopausal women for non-malignant gynecologic diseases at Northwestern University Prentice Women's Hospital such as cysts and fibroids (34–48 years old) at Northwestern University Prentice Women's Hospital. All of the samples were obtained from patients who were not taking hormonal or chemotherapy for at least 6 months prior to tissue collection and confirmed to be free of malignancy by histopathological examination (Suppl Table 4). The use of patient tissues was approved by the Institutional Review Board for Human Research (IRB) at Northwestern University and all patients provided written consent for the use of their tissue for research purposes. *Ex vivo* culture was established based on the published protocol [18] with modification. In brief, the fresh fimbriae tissues were incubated in a dissociation medium (DMEM, Life Technologies, Grand Island, NY) supplemented with 1.4 mg/ml Pronase (Roche Applied Science, Indianapolis, IN) and 0.1 mg/ml DNase (Sigma-Aldrich, St. Louis, MO) for 48 h at 4°C with constant mild agitation. The dissociated epithelial cells were harvested by centrifugation and re-suspended in DMEM/Ham's F12 1:1 (Life Technologies, Grand Island, NY) supplemented with 2% serum substitute, Ultroser G (PALL Life Sciences, Cergy-Saint-Christophe, France) and 1% penicillin/streptomycin (Life Technologies, Grand Island, NY). The FTE cells were transferred onto Primaria™ culture dishes (Corning Inc, Corning, NY) and incubated for a minimum of one hour to three hours to remove fibroblasts and red blood cells. Cells were then cultured on 60 mm dishes covered with collagen IV from human placenta (Sigma-Aldrich, St. Louis, MO) and incubated at 37°C in a humidified 5% CO_2_ incubator.

### Immortalized human FTSE cell lines and ovarian cancer cell lines

The FTE194 and FTE190 cells were immortalized by stable expressing human telomerase reverse transcriptase (hTERT) and SV40 large T antigen, which blocked the *p53* and *pRb* tumor suppressor pathways in primary FTSEs. The FTE237 and FTE246 cells were immortalized by stable expression of hTERT, *p53* shRNA, and CDK4^R24C^ in primary FTSEs [45] (Suppl Table 2). These cells were cultured in DMEM/Ham's F12 1:1 supplemented with 2% serum substitute and 1% penicillin/streptomycin.

### Quantitative real-time RT-PCR

Total RNA was isolated using the mirVana™ RNA Isolation Kit (Ambion, Austin, TX). The detailed method was described previously [22]. Then cDNA was prepared by a Mir-X™ miRNA First Strand Synthesis Kit (Clontech, Mountain View, CA). The entire sequence of mature miRNA was used as miRNA-specific, 5′ primer (Suppl Table 5A). The 3′ primer for qPCR is the mRQ 3′ Primer supplied with the kit. For real-time PCR, cDNA was synthesized by SYBR Green real-time PCRmaster mix (Life Technologies, Grand Island, NY) using The Applied Biosystems® StepOnePlus™ Real-Time PCR Systems (Applied Biosystems, Grand Island, NY) with sequence specific primers (Suppl Table 5B). Relative levels (ΔCt) were calculated by subtracting the cycle threshold (Ct) for internal control from the Ct. The mRNA and miRNA levels were normalized to GAPDH and U6, respectively. Results were obtained from at least three independent experiments performed in triplicate.

### Western blotting, immunofluorescence, and quantitative H2AX detection

#### Western blotting

Cultured cells were harvested and lysed on ice in a NP40 cell lysis buffer (Invitrogen, Grand Island, NY) supplemented with protease inhibitor cocktail (Thermo Scientific, Waltham, MA). Total protein (30 μg) was separated by SDS-PAGE and electro-transferred onto polyvinylidene fluoride membrane (Invitrogen, Grand Island, NY). The membrane was incubated with primary antibodies overnight at 4°C. The specific horseradish peroxidase-conjugated goat anti-rabbit or goat anti-mouse secondary antibody (Bio-Rad, Hercules, CA) was used to blot the target proteins (Suppl Table 6), and the secondary antibody was detected by an enhanced chemiluminescence ECL detection kit (Bio-Rad, Hercules, CA).

#### Immunofluorescence staining

Cells grown on coverslips (22 × 22 mm) were washed in PBS and fixed with 4% paraformaldehyde for 10 minutes. Cells were then permeabilized in 0.2% Triton X-100 for 10 minutes, blocked in 5% normal goat serum for 30 minutes, and then incubated with specific primary antibodies including mouse antihuman phospho-H2AX (1:200; EMD Millipore, Merck KGaA, Darmstadt, Germany) or mouse anti-human β-catenin (1:500, Santa Cruz Biotechnology, Dallas, TX) at 37°C for one hour. Mouse IgG was used as the negative control. After washing in PBS, cells were incubated with tetramethylrhodamine isothiocyanate-conjugated goat anti-mouse secondary antibody at room temperature for one hour. The nuclei were counterstained with 4,6-diamidino-2-phenylindole (DAPI). Three randomly selected fields of fluorescent images were captured under a fluorescence microscope (Delta Vision, Issaquah, WA), and a total of 100 cells were counted in each sample to calculate the percentage of positive-staining cells.

#### Quantitative H2AX detection

γ-H2AX was detected by ELISA using the HT γ-H2AX ELISA kit (Trevigen, Gaithersburg, MD) according to manufacturer's instructions. In brief, 2 × 10^6^ FTE cells were cultured in 60 mm petridish until 70–80% confluent. Total protein was extracted from cell pellets and added to 96 well plate coated with γ-H2AX antibody. Goat anti-Mouse IgM HRP Conjugate was used and results were taken by chemiluminescent readings using a Cytation 3 Cell Imaging Multi-Mode Reader (BioTek, Winooski, VT).

### miRNA profile

Total RNA was isolated by miRVana extraction kit (Ambion, Austin, TX). RT-PCR was performed by Megaplex without pre-amplification of miRNA method (Applied Biosystems, Grand Island, NY). The microarray assay was performed on a 384 miRNA probe (Megaplex RT-PCR product, Applied Biosystems, Grand Island, NY) and following the protocol of the manufacturer. The real-time data were analyzed by using SDS RQ manager software (Applied Biosystems, Grand Island, NY). Individual miRNA expression was analyzed by using individual miRNA primers specific for real-time PCR reactions.

### mRNA profile

Total RNA was isolated by mirVana™ method. Biotin labeled cRNA was generated from high quality total RNA. Purified second strand cDNA along with biotin UTPs were used to generate biotinylated, antisense RNA of each mRNA in an *in vitro* transcription (IVT) reaction. The labeled cRNAs were hybridized at 55°C overnight with the HumanHT-12 v4 Expression Beadchip (Illumina, San Diego, CA) and washed the following day. Signals was developed with Streptavidin-Cy3 and scanned with an Illumina iScan System. Quantile normalization and subsequent data processing were performed using the GeneSpring GX v13.0 software package (Agilent Technologies, Santa Clara, CA). DAVID Bioinformatics Resources 6.7 was used for pathway analysis. The microarray data have been deposited in NCBI GEO database under the accession number GSE (pending).

### Stable and transient miRNA and si-RNA gene expression

Anti-*miR-182* and scramble control were from Regulus Therapeutics. The *si-β-catenin* and *si-P21* were purchased from Life Technologies. The transient transfections for miRNA mimic and anti-*miR-182* were previously described [22]. Pre-*miR-182* lentivirus was prepared as previously described and its stable overexpression was made in FTE190, FTE194, FTE237, and FTE246 cell lines by the method described previously [23]. *sh-p53* pLKO.1 was obtained from Addgene (Cambridge, MA). *Ad-S37A-β-cat-HA* was provided by Jan Kitajewski at Columbia University, New York.

### Senescence stain and quantitative detection

#### Senescence-associated β-Gal stain

Senescence-associated β-Gal stain were used as previously described [16]. In brief, cells were seeded onto coverslips, placed in 6-well plates overnight, and then treated with the test compounds: H_2_O_2_ (100 or 200 μM), or DOX (0.2 μg/mL) for 48 hrs. Cells were fixed with 2% formaldehyde and 0.2% glutaraldehyde in PBS at room temperature for 5 minutes. After washing in PBS, cells were stained with staining solution containing 1 mg/mL X-gal (Life Technologies, Grand Island, NY) and incubated in a CO_2_-free incubator at 37°C for 16 hours. Blue cells were counted under the microscope and statistically analyzed. Three randomly selected fields (1 × 1 mm^2^) of images were captured to count the senescence rate (%).

#### Quantitative detection for senescence

Cellular senescence was measured by fluorimetric beta-galactosidase activity using Cellular Senescence Assay Kit (Cell Biolabs Inc. San Diego, CA). In brief, cells were seeded on 6-well plates overnight and then treated with the test compounds: H_2_O_2_ (100 μM), or DOX (0.2 μg/mL) for 48 hrs. At the end of treatment, the cell lysates were prepared for the determination of SA-β-galactosidase activity using the fluorescence plate reader at 360/465 nm. Cell lysate protein concentration was determined for normalization cell number and expressed as RLU/cell number.

### Chronic ROS stress

The chronic ROS stress on the immortalized FTSE cells was performed by reapplication of a low dose of H_2_O_2_. In brief, FTSE cells including FTE190, FTE194, FTE237 and FT246, with and without stable miR-182 overexpression were cultured in 100 mm dishes and pulse treated with 10 μM H_2_O_2_ for three times per week. This low dose treatment lasted for 15 passages then the H_2_O_2_ concentration was increased to 100 μM for an additional 2–3 passages.

### Soft agar colony-formation assay

Soft agar colony formation assay was performed as previously described [22]. The cells (0.75 × 10^4^ cells) were suspended in 3 mL of culture medium containing 0.3% agar (USB Corporation, OH, USA) and seeded onto a base layer of 3 mL of a 0.6% agar bed in 60-mm tissue culture dishes. After up to 6 weeks, colonies were stained with 0.005% crystal violet and photographed. Colonies > 0.1 mm in diameter were counted, and the average numbers (*n* = 3) in each group were calculated.

### Xenograft in nude mice

Senescence bypass cells for FTSE cells with and without *miR-182* overexpression were engrafted in intraovarian bursa of female nude mice (NCI-Frederick) as previously described [22]. The xenograft growth was monitored by IVIS weakly. Experiments were approved by the Institutional Animal Care and Use Committee of Northwestern University.

### Cytogenetic analysis

Cells were grown in 60 mm dishes and KaryoMAX® Colcemid (10 μl/mL, Life Technologies, Grand Island, NY) was added when cells reached 85% confluence, incubating for 2 hours and rinsed with PBS. Then cells were suspended by 2 mL 0.25% trypsin (Life Technologies, Grand Island, NY) and centrifuged. The pre-warmed hypotonic solution (0.075 M potassium chloride and 0.9% sodium citrate, 1:1) was added and cells were incubated at 37°C, for 16 min. Cells were fixed in fixation buffer (methanol: glacial acetic acid, 3:1) overnight and dropped on ice-cold slide. Slides were stained by Giemsa and photographed in oil lenses for karyotype analysis.

### FACS sorting for fallopian tube secretory (FTSE) and ciliated (FTCE) cells

Primary FTE cells were isolated and cultured up to two weeks. Single cell suspensions were prepared in HBSS buffer and labeled with mouse anti-human LhS28-APC antibody (Novus, Littleton, CO), which recognizes the basal bodies of cilia in ciliated epithelial cells [47], and Pacific Blue mouse anti-human CD326 (EpCAM) (epithelial marker) antibody (BioLegend, San Diego, CA), a marker for epithelial cells [48]. The cells were sorted using BD FACSAria 5-Laser Flow Cytometry (BD Biosciences, Franklin Lakes, NJ) with 488 nm and 633 nm lasers for excitation. Statistics and images were then created offline using FCS EXPRESS VERSION 3 (De Novo Software, Glendale, CA). The sorted cells were collected in DMEM/F12 medium with 2% Ultroser G and cultured for the related assays.

### Human and bovine follicular fluid

The use of human follicular fluid (h-FF) was approved by IRB at Northwestern University. Four h-FF samples were obtained from donors undergoing oocyte retrieval as part of their *in vitro* fertilization procedure at Northwestern Hospital IVF laboratory with a written informed patient consent. Bovine follicular fluid (b-FF) was acquired from the Aurora Packing Company (Aurora, IL, USA). Follicular fluid was centrifuged at 500 g for 10 min at 4°C, and the supernatant was aliquotted and stored at −80°C until use. Both of h-FF and b-FF were heated to 56°C for 30 min prior to use.

### ROS detection and measurement

Intracellular ROS levels in human fallopian tube epithelial (FTE) cells were measured using the dye, dihydroethidium (DHE) (Molecular Probe Inc., Eugene, OR) by immunofluorescence microscope imaging and flow cytometry [49].

For ROS detection using fluorescence microscope imaging, primary *ex vivo* culture FTE cells (1 × 10^5^ cells) were treated with h-FF or H_2_O_2_ for 4–24 hours, FTE cells were loaded with DHE (10 μM) and incubated at 37°C in the dark for 30 min. The fluorescent images were captured immediately with a Nikon C2+ Confocal Microscope System (Nikon, Melville, NY) at excitation and emission wavelengths of 520 and 610 nm, respectively.

For ROS detection using flow cytometry, FTE cells (5 × 10^5^) were cultured on 60-mm Petri dishes. After treatment with h-FF or b-FF and/or 5 mM NAC for 2 or 24 hours, FTE cells were loaded with DHE (10 μM) for 30 min at 37°C in the dark. Pulse treatment with 100 μM of H2O2 (3 times with 1 hour interval) served as positive control. The dosage of H2O2 being used is according to clonogenic cell killing experiment and references (Data not shown). A total of 1 × 10^4^ cells were analyzed within 60 min of staining by a FASC caliber flow cytometer (BD LSRFortessa Analyzer, BD Biosciences, Franklin Lakes, NJ). The relative increase in ROS was calculated by comparing the mean fluorescence intensity of DHE stained-treatment groups to that of the untreated group.

### Statistical analysis

All data are presented as means and standard errors of at least three independent experiments. The Student *t* test was used for comparisons between two groups of experiments, and one-way ANOVA analysis was used for comparisons among three or more groups. Statistical tests were two-sided and *P* < 0.05 was considered statistically significant.

## SUPPLEMENTARY TABLES AND FIGURES



## References

[1] Kurman RJ, Shih Ie M (2010). The origin and pathogenesis of epithelial ovarian cancer: a proposed unifying theory. Am J Surg Pathol.

[2] Cancer-Genome-Atlas-Research-Network (2011). Integrated genomic analyses of ovarian carcinoma. Nature.

[3] Wei JJ, Wu J, Luan C, Yeldandi A, Lee P, Keh P, Liu J (2010). HMGA2: a potential biomarker complement to P53 for detection of early-stage high-grade papillary serous carcinoma in fallopian tubes. Am J Surg Pathol.

[4] Bowtell DD (2010). The genesis and evolution of high-grade serous ovarian cancer. Nat Rev Cancer.

[5] Perets R, Wyant GA, Muto KW, Bijron JG, Poole BB, Chin KT, Chen JY, Ohman AW, Stepule CD, Kwak S, Karst AM, Hirsch MS, Setlur SR, Crum CP, Dinulescu DM, Drapkin R (2013). Transformation of the fallopian tube secretory epithelium leads to high-grade serous ovarian cancer in brca;tp53;pten models. Cancer cell.

[6] Magenta A, Greco S, Gaetano C, Martelli F (2013). Oxidative stress and microRNAs in vascular diseases. International journal of molecular sciences.

[7] Revelli A, Delle Piane L, Casano S, Molinari E, Massobrio M, Rinaudo P (2009). Follicular fluid content and oocyte quality: from single biochemical markers to metabolomics. Reproductive biology and endocrinology: RB&E.

[8] Suzuki HI, Yamagata K, Sugimoto K, Iwamoto T, Kato S, Miyazono K (2009). Modulation of microRNA processing by p53. Nature.

[9] Karst AM, Levanon K, Drapkin R (2011). Modeling high-grade serous ovarian carcinogenesis from the fallopian tube. Proc Natl Acad Sci U S A.

[10] Medeiros F, Muto MG, Lee Y, Elvin JA, Callahan MJ, Feltmate C, Garber JE, Cramer DW, Crum CP (2006). The tubal fimbria is a preferred site for early adenocarcinoma in women with familial ovarian cancer syndrome. Am J Surg Pathol.

[11] Levanon K, Crum C, Drapkin R (2008). New insights into the pathogenesis of serous ovarian cancer and its clinical impact. Journal of clinical oncology: official journal of the American Society of Clinical Oncology.

[12] Jordan SJ, Green AC, Whiteman DC, Moore SP, Bain CJ, Gertig DM, Webb PM (2008). Serous ovarian, fallopian tube and primary peritoneal cancers: a comparative epidemiological analysis. Int J Cancer.

[13] Agarwal A, Gupta S, Sharma RK (2005). Role of oxidative stress in female reproduction. Reproductive biology and endocrinology: RB&E.

[14] Adams PD (2009). Healing and hurting: molecular mechanisms, functions, and pathologies of cellular senescence. Mol Cell.

[15] Song Z, Liu Y, Hao B, Yu S, Zhang H, Liu D, Zhou B, Wu L, Wang M, Xiong Z, Wu C, Zhu J, Qian X (2014). Ginsenoside Rb1 prevents H2O2-induced HUVEC senescence by stimulating sirtuin-1 pathway. PloS one.

[16] Xu X, Lu Z, Qiang W, Vidimar V, Kong B, Kim JJ, Wei JJ (2014). Inactivation of AKT induces cellular senescence in uterine leiomyoma. Endocrinology.

[17] Zavadil J, Ye H, Liu Z, Wu J, Lee P, Hernando E, Soteropoulos P, Toruner GA, Wei J-J (2010). Profiling and functional analyses of microRNAs and their target gene products in human uterine leiomyomas. PloS one.

[18] Levanon K, Ng V, Piao HY, Zhang Y, Chang MC, Roh MH, Kindelberger DW, Hirsch MS, Crum CP, Marto JA, Drapkin R (2010). Primary *ex vivo* cultures of human fallopian tube epithelium as a model for serous ovarian carcinogenesis. Oncogene.

[19] He X, Duan C, Chen J, Ou-Yang X, Zhang Z, Li C, Peng H (2009). Let-7a elevates p21(WAF1) levels by targeting of NIRF and suppresses the growth of A549 lung cancer cells. FEBS letters.

[20] Zhao J, Lammers P, Torrance CJ, Bader AG (2013). TP53-independent function of miR-34a via HDAC1 and p21(CIP1/WAF1.). Molecular therapy : the journal of the American Society of Gene Therapy.

[21] Li XL, Hara T, Choi Y, Subramanian M, Francis P, Bilke S, Walker RL, Pineda M, Zhu Y, Yang Y, Luo J, Wakefield LM, Brabletz T, Park BH, Sharma S, Chowdhury D (2014). A p21-ZEB1 complex inhibits epithelial-mesenchymal transition through the microRNA 183-96-182 cluster. Molecular and cellular biology.

[22] Xu X, Ayub B, Liu Z, Serna VA, Qiang W, Liu Y, Hernando E, Zabludoff S, Kurita T, Kong B, Wei JJ (2014). Anti-miR182 reduces ovarian cancer burden, invasion, and metastasis: an *in vivo* study in orthotopic xenografts of nude mice. Molecular cancer therapeutics.

[23] Liu Z, Liu J, Segura MF, Shao C, Lee P, Gong Y, Hernando E, Wei JJ (2012). MiR-182 overexpression in tumourigenesis of high-grade serous ovarian carcinoma. The Journal of pathology.

[24] Yao E, Ventura A (2011). A new role for miR-182 in DNA repair. Mol Cell.

[25] Emori MM, Drapkin R (2014). The hormonal composition of follicular fluid and its implications for ovarian cancer pathogenesis. Reproductive biology and endocrinology: RB&E.

[26] Balatti V, Maniero S, Ferracin M, Veronese A, Negrini M, Ferrocci G, Martini F, Tognon MG (2011). MicroRNAs Dysregulation in Human Malignant Pleural Mesothelioma. J Thorac Oncol.

[27] Moskwa P, Buffa FM, Pan Y, Panchakshari R, Gottipati P, Muschel RJ, Beech J, Kulshrestha R, Abdelmohsen K, Weinstock DM, Gorospe M, Harris AL, Helleday T, Chowdhury D (2011). miR-182-mediated downregulation of BRCA1 impacts DNA repair and sensitivity to PARP inhibitors. Mol Cell.

[28] Peng X, Li W, Yuan L, Mehta RG, Kopelovich L, McCormick DL (2013). Inhibition of Proliferation and Induction of Autophagy by Atorvastatin in PC3 Prostate Cancer Cells Correlate with Downregulation of Bcl2 and Upregulation of miR-182 and p21. PloS one.

[29] Vurusaner B, Poli G, Basaga H (2012). Tumor suppressor genes and ROS: complex networks of interactions. Free radical biology & medicine.

[30] Wu J, Lu LY, Yu X (2010). The role of BRCA1 in DNA damage response. Protein Cell.

[31] Tsai WB, Chung YM, Takahashi Y, Xu Z, Hu MC (2008). Functional interaction between FOXO3a and ATM regulates DNA damage response. Nat Cell Biol.

[32] Guttilla IK, White BA (2009). Coordinate regulation of FOXO1 by miR-27a, miR-96, and miR-182 in breast cancer cells. J Biol Chem.

[33] Myatt SS, Wang J, Monteiro LJ, Christian M, Ho KK, Fusi L, Dina RE, Brosens JJ, Ghaem-Maghami S, Lam EW (2010). Definition of microRNAs that repress expression of the tumor suppressor gene FOXO1 in endometrial cancer. Cancer Res.

[34] Segura MF, Hanniford D, Menendez S, Reavie L, Zou X, Alvarez-Diaz S, Zakrzewski J, Blochin E, Rose A, Bogunovic D, Polsky D, Wei J, Lee P, Belitskaya-Levy I, Bhardwaj N, Osman I (2009). Aberrant miR-182 expression promotes melanoma metastasis by repressing FOXO3 and microphthalmia-associated transcription factor. Proc Natl Acad Sci U S A.

[35] Chiang CH, Hou MF, Hung WC (2013). Up-regulation of miR-182 by beta-catenin in breast cancer increases tumorigenicity and invasiveness by targeting the matrix metalloproteinase inhibitor RECK. Biochimica et biophysica acta.

[36] McMillen BD, Aponte MM, Liu Z, Helenowski IB, Scholtens DM, Buttin BM, Wei JJ (2012). Expression analysis of MIR182 and its associated target genes in advanced ovarian carcinoma. Modern pathology: an official journal of the United States and Canadian Academy of Pathology, Inc.

[37] Kinross KM, Brown DV, Kleinschmidt M, Jackson S, Christensen J, Cullinane C, Hicks RJ, Johnstone RW, McArthur GA (2011). *In vivo* activity of combined PI3K/mTOR and MEK inhibition in a Kras(G12D);Pten deletion mouse model of ovarian cancer. Molecular cancer therapeutics.

[38] Kim J, Coffey DM, Creighton CJ, Yu Z, Hawkins SM, Matzuk MM (2012). High-grade serous ovarian cancer arises from fallopian tube in a mouse model. Proc Natl Acad Sci U S A.

[39] Ambekar AS, Nirujogi RS, Srikanth SM, Chavan S, Kelkar DS, Hinduja I, Zaveri K, Prasad TS, Harsha HC, Pandey A, Mukherjee S (2013). Proteomic analysis of human follicular fluid: a new perspective towards understanding folliculogenesis. Journal of proteomics.

[40] Coussens LM, Werb Z (2002). Inflammation and cancer. Nature.

[41] Lu H, Ouyang W, Huang C (2006). Inflammation, a key event in cancer development. Molecular cancer research: MCR.

[42] Murdoch WJ, Martinchick JF (2004). Oxidative damage to DNA of ovarian surface epithelial cells affected by ovulation: carcinogenic implication and chemoprevention. Experimental biology and medicine.

[43] Folkins AK, Jarboe EA, Saleemuddin A, Lee Y, Callahan MJ, Drapkin R, Garber JE, Muto MG, Tworoger S, Crum CP (2008). A candidate precursor to pelvic serous cancer (p53 signature) and its prevalence in ovaries and fallopian tubes from women with BRCA mutations. Gynecologic oncology.

[44] Lee Y, Miron A, Drapkin R, Nucci MR, Medeiros F, Saleemuddin A, Garber J, Birch C, Mou H, Gordon RW, Cramer DW, McKeon FD, Crum CP (2007). A candidate precursor to serous carcinoma that originates in the distal fallopian tube. The Journal of pathology.

[45] Karst AM, Drapkin R (2012). Primary culture and immortalization of human fallopian tube secretory epithelial cells. Nature protocols.

[46] Wu J, Liu Z, Shao C, Gong Y, Hernando E, Lee P, Narita M, Muller W, Liu J, Wei JJ (2011). HMGA2 overexpression-induced ovarian surface epithelial transformation is mediated through regulation of EMT genes. Cancer Res.

[47] Comer MT, Andrew AC, Leese HJ, Trejdosiewicz LK, Southgate J (1999). Application of a marker of ciliated epithelial cells to gynaecological pathology. Journal of clinical pathology.

[48] Shultz LD, Saito Y, Najima Y, Tanaka S, Ochi T, Tomizawa M, Doi T, Sone A, Suzuki N, Fujiwara H, Yasukawa M, Ishikawa F (2010). Generation of functional human T-cell subsets with HLA-restricted immune responses in HLA class I expressing NOD/SCID/IL2r gamma(null) humanized mice. Proc Natl Acad Sci U S A.

[49] Qiang W, Cahill JM, Liu J, Kuang X, Liu N, Scofield VL, Voorhees JR, Reid AJ, Yan M, Lynn WS, Wong PK (2004). Activation of transcription factor Nrf-2 and its downstream targets in response to moloney murine leukemia virus ts1-induced thiol depletion and oxidative stress in astrocytes. Journal of virology.

