# Supplementary material for: Role of miR-182 in response to oxidative stress in the cell fate of human fallopian tube epithelial cells
Source: Oncotarget. 2015 Oct 12;6(36):38983–98. doi: 10.18632/oncotarget.5493 (PMC4770751; doi:10.18632/oncotarget.5493)
Supplement: Supplementary file 1 [file oncotarget-06-38983-s001.pdf]

SUPPLEMENTARY TABLES AND FIGURES

Supplementary Table S1: ROS induced miRNA expression in primary FTE cells

miRNA	Fold change	miRNA	Fold change
hsa-miR-10b	24.16953	*hsa-miR-502-3p*	0.16011
hsa-miR-222	14.13471	*hsa-miR-33b*	0.05828
hsa-let-7d	4.96440	*hsa-miR-492*	0.04320
hsa-miR-29b	4.72215	*hsa-miR-433*	0.00309
hsa-let-7f	3.32550		
hsa-miR-34a	3.31579		
hsa-miR-34c	3.15606		
hsa-miR-21	3.05234		
hsa-miR-205	2.76332		
hsa-miR-449b	2.44541		
hsa-miR-10a	2.30499		
hsa-miR-29a	2.17290		
hsa-miR-200b	2.14990		
hsa-let-7e	2.09707		
hsa-let-7a	2.05897		
hsa-let-7g	2.05270		
hsa-miR-29c	1.92444		
hsa-let-7b	1.88443		
hsa-miR-182	1.64113		

Supplementary Table S2: Normal and malignant cell lines used in this study

Cell type	Gene phenotype	Vendor and source
FTE194, FTE190	human telomerase reverse transcriptase (hTERT) and SV40 large T antigen	Dr. Drapkin
FTE237, FTE246	hTERT, p53 shRNA and $CDK4^{R24C}$	Dr. Drapkin
FTE194-miR-182	FT194 transfected with lenti-miR-182	
FTE237-miR-182	FT237 transfected with lenti-miR-182	
SKOV3	Human ovarian cancer cells derived from ascite fluid?	ATCC
OVCAR3	high endogenous miR-182	ATCC

Supplementary Table S3A: mRNA profile in primary fallopian tube epithelial cells between control and ROS (100 µM) treated Cells (Fold change > 1.5)

Pathway	*P* Value	Genes	Regulation	Fold change	*P* value
Cell Cycle	1.62E-08	MCM2	down	1.631111	0.009494
		MCM3	down	1.715614	0.009944
		MCM6	down	1.807582	0.024181
		MCM7	down	1.812217	0.024483
		CDK2	down	1.505568	0.022419
		BUB1B	down	1.548273	0.020757
		CDC45L	down	2.396634	0.011079
		E2F2	down	2.429783	0.034599
		CDC2	down	1.889084	0.037122
		CDKN1A	up	2.785754	5.73E-04
DNA Replication	3.44E-08	MCM2	down	1.631111	0.009494
		MCM3	down	1.715614	0.009944
		MCM6	down	1.807582	0.024181
		MCM7	down	1.812217	0.024483
		POLA2	down	1.668838	8.59E-04
		FEN1	down	1.968111	0.04464
		RFC4	down	1.506162	0.010166
p53 signaling pathway	0.00120142	CDK2	down	1.505568	0.022419
		SESN1	down	1.534391	0.028517
		CDKN1A	up	2.785754	5.73E-04
		CDC2	up	1.889084	0.037122
Pathways in Cancer	0.00921557	CDK2	down	1.505568	0.022419
		MSH6	down	1.619989	6.45E-05
		CKS1B	down	1.746261	0.040697
		E2F2	down	2.429783	0.034599
		BCL2L1	up	1.545341	0.022417
		ITGA2	up	1.509473	0.03877
		CDKN1A	up	2.785754	5.73E-04

Supplementary Table S3B: Net gain and loss of ROS induced mRNA in primary FTE cells

SYMBOL	FC (abs)	*p* values	Regulation
GDF15	9.08	0.0001	up
CDKN1A	2.79	0.0006	up
HMOX1	2.56	0.0402	up
LOC643031	2.37	0.0054	up
PHLDA3	2.01	0.0002	up
PPP1R15A	2.00	0.0017	up
MIR1978	1.99	0.0129	up
RRAD	1.85	0.0005	up
MT1M	1.78	0.0264	up
DUSP5	1.75	0.0098	up
RRAGC	1.74	0.0055	up
NUPR1	1.68	0.0420	up
LOC100008588	1.67	0.0213	up
KLHL21	1.66	0.0141	up
LOC387763	1.64	0.0497	up
TXNRD1	1.63	0.0369	up
P8	1.57	0.0459	up
AKR1C4	1.56	0.0419	up
BCL2L1	1.55	0.0224	up
TRIB1	1.54	0.0136	up
SESN1	1.53	0.0285	up
SPATA18	1.53	0.0093	up
UBAP1	1.53	0.0064	up
SRXN1	1.53	0.0382	up
STOM	1.52	0.0160	up
ITGA2	1.51	0.0388	up
PANX2	1.50	0.0380	up
DNER	1.50	0.0378	up
CDK2	−1.51	0.0224	down
RFC4	−1.51	0.0102	down
SKA2	−1.52	0.0116	down
ATAD2	−1.52	0.0038	down
ITGB3BP	−1.53	0.0112	down
PARP1	−1.53	0.0104	down

SYMBOL	FC (abs)	*p* values	Regulation
ECT2	−1.54	0.0013	down
BUB1B	−1.55	0.0208	down
LMNB1	−1.55	0.0448	down
SUV39H1	−1.55	0.0118	down
KPNA2	−1.56	0.0432	down
NT5E	−1.56	0.0301	down
DTL	−1.56	0.0077	down
WDR51A	−1.56	0.0141	down
UBE2T	−1.57	0.0378	down
KNTC1	−1.57	0.0197	down
CHAF1B	−1.61	0.0003	down
FANCI	−1.61	0.0115	down
MCM10	−1.61	0.0107	down
MSH6	−1.62	0.0001	down
MYH10	−1.62	0.0471	down
FANCG	−1.63	0.0071	down
MCM2	−1.63	0.0095	down
GMNN	−1.66	0.0065	down
POLA2	−1.67	0.0009	down
ARHGDIB	−1.69	0.0409	down
KIF22	−1.67	0.0384	down
MCM3	−1.72	0.0099	down
FBXO5	−1.73	0.0134	down
CKS1B	−1.75	0.0407	down
POLQ	−1.75	0.0166	down
BARD1	−1.79	0.0330	down
MCM6	−1.81	0.0242	down
MCM7	−1.81	0.0245	down
DNMT1	−1.82	0.0073	down
MELK	−1.84	0.0290	down
CDC2	−1.89	0.0371	down
HIST1H4C	−1.92	0.0156	down
FEN1	−1.97	0.0446	down
ASF1B	−2.02	0.0489	down
CDCA7	−2.04	0.0038	down

(*Continued*)

SYMBOL	FC (abs)	*p* values	Regulation
PLK4	−2.05	0.0130	down
RAD51AP1	−2.14	0.0013	down
CDCA5	−2.18	0.0413	down
CDT1	−2.24	0.0081	down
UHRF1	−2.35	0.0214	down
CDC45L	−2.40	0.0111	down
E2F2	−2.43	0.0346	down
GINS2	−2.54	0.0201	down

Supplementary Table S4: Human Fallopian tube tissue sample used for this study

Deidentification No.	patient's age	tissue type	Indication for surgery
FT709	41	Fallopian tube Fimbria	Fibroids
FT1023	34	Fallopian tube Fimbria	Fibroids
FT1121	45	Fallopian tube Fimbria	Fibroids
FT110	45	Fallopian tube Fimbria	Uterine Prolapse
FT116	40	Fallopian tube Fimbria	Dysfunction uterine bleeding
FT313	41	Fallopian tube Fimbria	Fibroids
FT319	48	Fallopian tube Fimbria	Endometriosis
FT325	42	Fallopian tube Fimbria	Fibroids
FT414	42	Fallopian tube Fimbria	Fibroids
FT505	46	Fallopian tube Fimbria	Fibroids

Supplementary Table S5: Primer sequences for miRNA and mRNA

A. miRNA

miRNA name	Primer sequences
miR-182	5′-TTTGGCAATGGTAGAACTCACACT-3′
miR-183	5′-TATGGCACTGGTAGAATTCACT-3′
miR-96	5′-TTTGGCACTAGCACATTTTTGCT-3′
miR-34a	5′-TGGCAGTGTCTTAGCTGGTTGT-3′
miR-34c	5′-AGGCAGTGTAGTTAGCTGATTGC-3′
miR-29b	5′-TAGCACCATTTGAAATCAGTGTT-3′
miR-200a	5′-TAGCACCATTTGAAATCAGTGTT-3′
miR-200c	5′-TAATACTGCCGGGTAATGATGGA-3′
let-7d	5′-AGAGGTAGTAGGTTGCATAGTT-3′
miR-502-3p	5′-AATGCACCTGGGCAAGGATTCA-3′
miR-33b	5′-GTGCATTGCTGTTGCATTGC-3′

B. gene

gene name	Forward	Reverse
GAPDH	5′-TGCACCACCAACTGCTTAGC-3′	5′-GGCATGGACTGTGGTCATGAG-3′
β-catenin	5′-GCGCCATTTTAAGCCTCTCG-3′	5′-GAGTAGCCATTGTCCACGCT-3′
IL-6R	5′-ATCCCTGACGACAAAGGCTG-3′	5′-CTGAACTTGCTCCCGACACT-3′
p53	5′-AGCCAAGTCTGTGACTTGCA-3′	5′-AACCTCCGTCATGTGCTGT-3′
PAX8	5′-TCAACCTCCCTATGGACAGC-3′	5′-GCCTCGCTGTAGGAGGAGTA-3′
TNFαIP2	5′-GTCTGCCTTTTAACGGTCTG-3′	5′-TGGGGAGTGAATAAGAGGGT-3′
BCL2	5′-CTGCACCTGACGCCCTTCACC-3′	5′-CACATGACCCCACCGAACTCAAAGA-3′
FOXJ1	5′-CCAAGAATTGGCAAAAGCA-3′	5′-TTTCTCTGGGTAGGGACCTG-3′
SAL2	5′-CACGAATCCGAGAGGAGCTCTC-3′	5′-CACCATTACAGGAGGGTCAGTAG-3′
SELENBP1	5′-TCAGATGATCCAGCTCAGCCT-3′	5′-TCACAGAGCCTTCCCTGATGA-3′
Notch1	5′-GAGGCGTGGCAGACTATGC-3′	5′-CTTGTACTCCGTCAGCGTGA-3′
Pre-miR-182	5′-CCTAGCGTTAACCTCCTCTTGGCAGCACCCCT-3′	5′-GATCCGCTCGAGCGACCCTGCAGG AAGGACCT-3′
pri-miR-182	5′-CGGAATTCGGAAGGACCTTGTCGCAGTTGC-3′	5′-GACACTCGAGCCAGTTCCCTCACT CCTCGA-3′

Supplementary Table S6: Antibodies used in this study

antibody	vendor	working concentration		
		WB	IF	Flow Cytometry
BRCA1	EMD Millipore	1:1000		
β-catenin	Santa Cruz	1:2000	1:500	
P53	Santa Cruz	1:2000		
P21	Cell Signaling Technology	1:2000		
β-actin	Sigma	1:5000		
Phospho-H2AX	Upstate		1:200	
LhS28-APC	Novus Biologicals		1:200	1:200

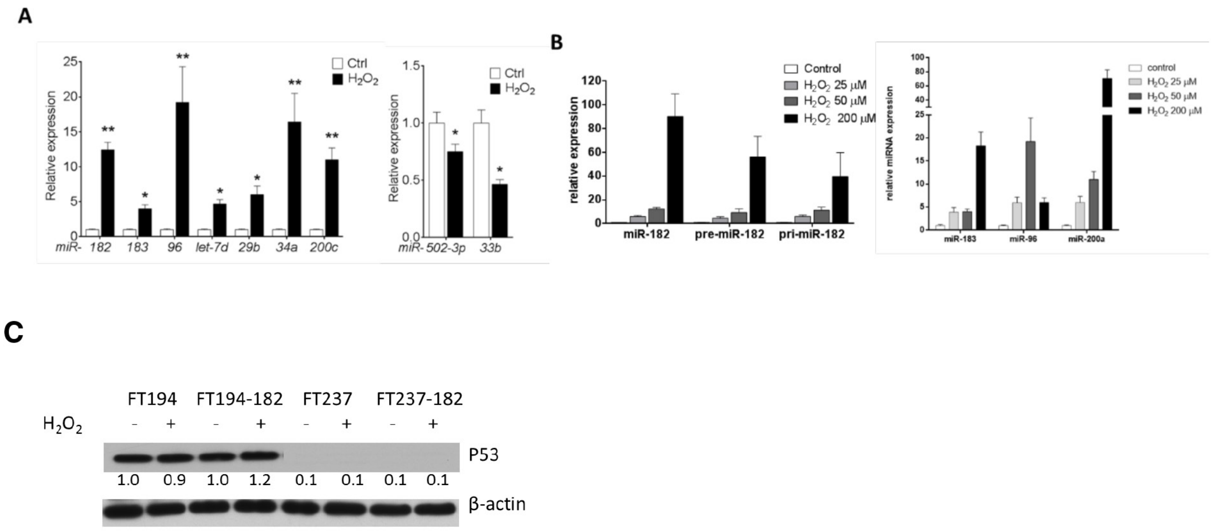

Supplementary Figure S1: ROS induced miRNA (ROSmiRs) and mRNA expression in FTE cells. **A.** Validation of ROS-induced miRNA expression (black box) was examined by real-time RT-PCR in primary fallopian tube epithelial cells. **B.** ROSmiR expression in different doses of H_2O_2 treatment in FTE194 cell line. **C.** Western blot analysis of TP53 expression in immortalized fallopian tube secretory epithelial cells FTE194 and FTE237. β-actin was used as a protein loading control.

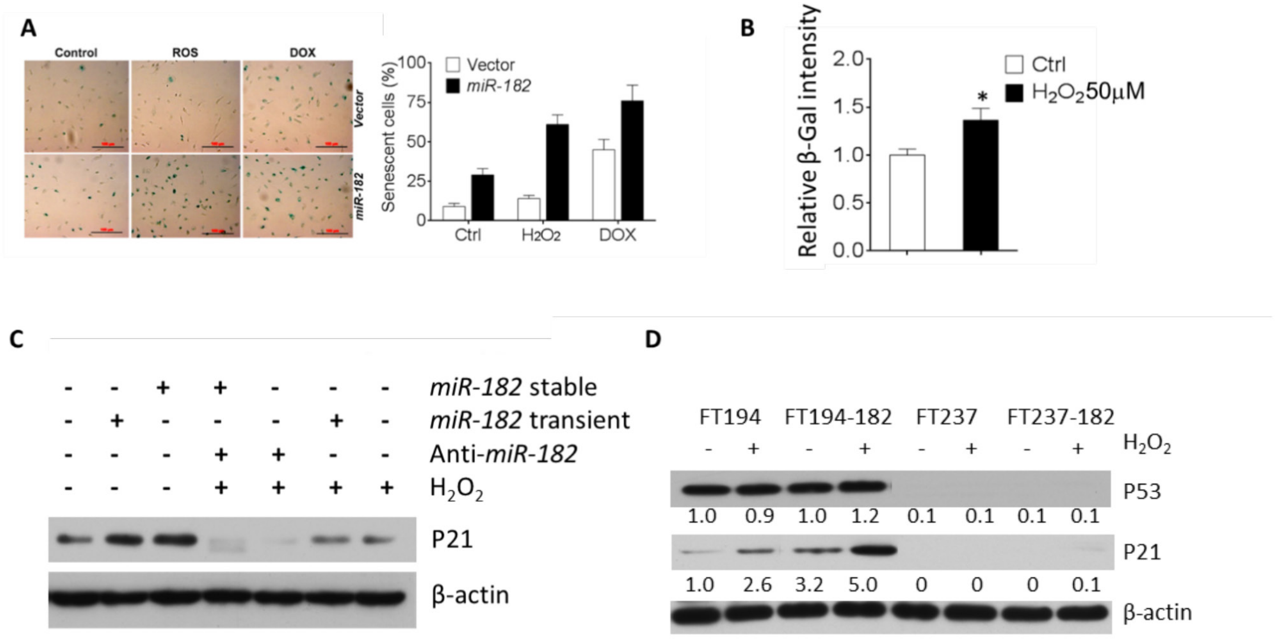

Supplementary Figure S2: ROS induced premature cellular senescence is mediated by ROSmiRs and cell cycle genes. A. Senescence analysis by SA-β-Gal stain in FTE194 cell lines treated by ROS (100 μM H_2O_2) or DOX (0.25 μg/ml) and senescence rate in cells with and without *miR-182* overexpression. **B.** Quantitative analysis of β-Gal stain intensity in liquid-based measurement. **C.** Western blot analysis of P21 expression in FTE194 cell line by controlling *miR-182* (stable by lentiviral *miR-182*, and transient transfection of 60 nM *miR-182* mimic) and with or without ROS (H_2O_2) exposure. **D.** P21 and P53 expression in FTSE cells of FTE194 and FTE237 with and without miR-182 expression.

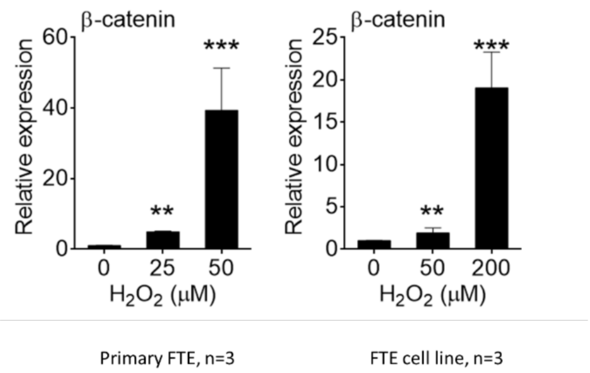

Supplementary Figure S3: ROS or stress-induced miR-182 expression is regulated by β-catenin. Quantitative analysis of ROS-induced *β-catenin* expression in primary (left) and immortalized (right) fallopian tube epithelial cells detected by real-time RT-PCR. The data were obtained in three different samples (*n* = 3) for each. Primary FTE cells are more sensitive to ROS-induced β-catenin.

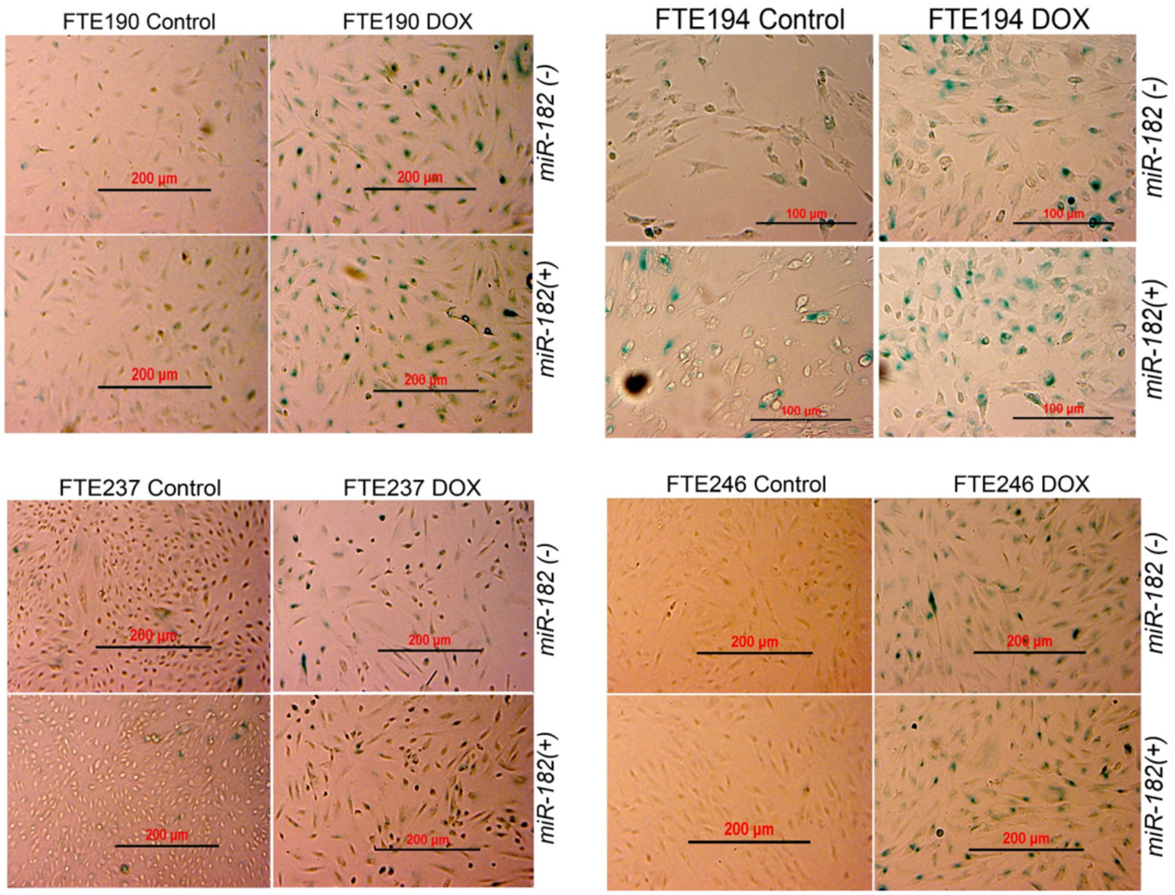

Supplementary Figure S4: miR-182 enhances senescence bypass in FTSE cells under DNA stress and impaired p53. Photomicrographs illustrating senescence β-Gal stain in four immortalized fallopian tube secretory epithelial cells with *(miR-182(+))* and without *(miR-182(-)) miR-182 overexpression treated with saline (Control) or Doxorubicin (DOX, 0.25 µg/ml)*.

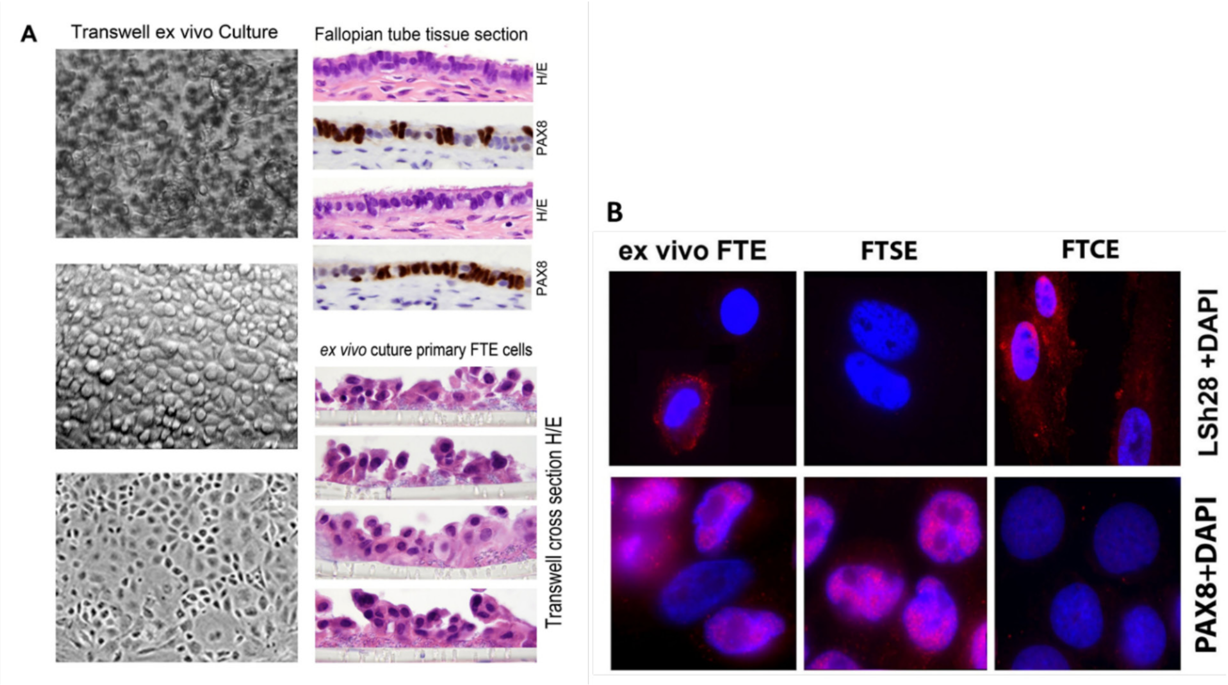

Supplementary Figure S5: Differential reaction to ROS and DNA damage response between FTSE and FTCE cells. A. Photomicrographs illustrate some examples of *ex vivo* culture of primary human fallopian tube epithelial cells in inverted microscope and transwell H/E section. **B.** Immunofluorescent stain of PAX8 and LhS28 in primary fallopian tube epithelial cells of non-sorted (FTE) and sorted secretory (FTSE) and ciliated (FTCE) cell. DAPI was used as counter stain.

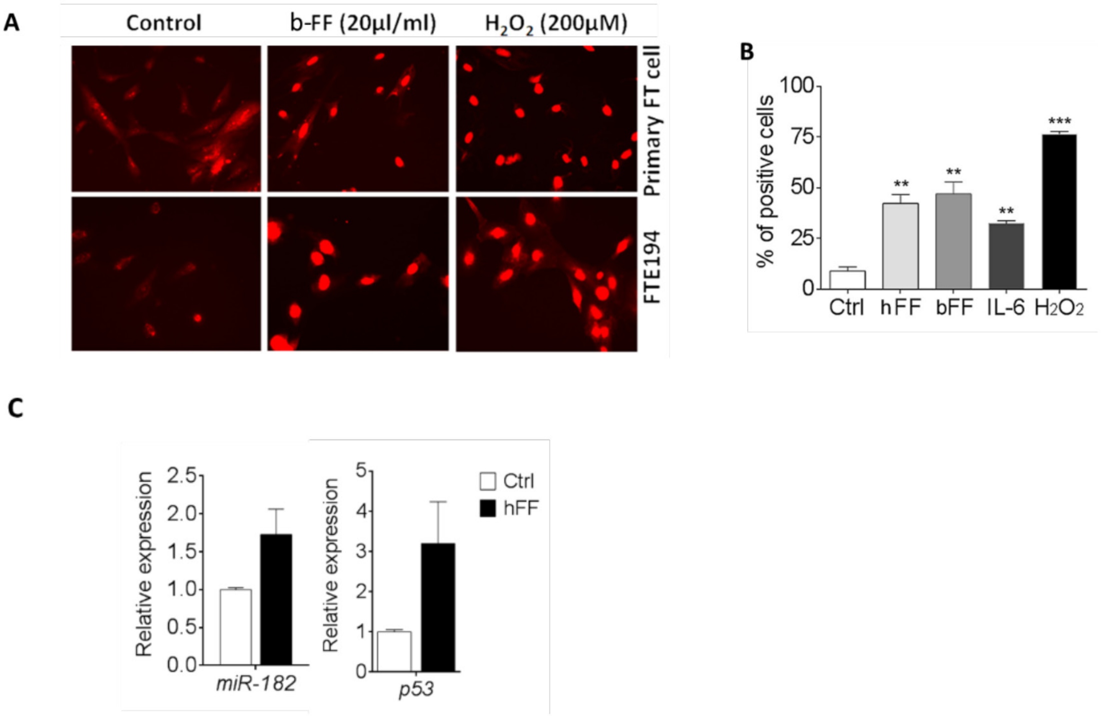

Supplementary Figure S6: Follicular fluid and interleukins for ROS induction in fallopian tube epithelial cells. A. Photomicrographs show intracytoplasmic ROS production induced by bovine follicular fluid (b-FF) and ROS control human follicular fluid (h-FF) and ROS control in the primary FTE and immortalized cell line. **B.** Histograph illustrates the percentage of DHE stained positive cells in control (Ctrl), human (h-FF), bovine (b-FF), interleukin-6 (IL-6) and positive control (H_2O_2) treated cells. **C.** *miR-182* and *p53* expression in primary FTE cells treated with human follicular fluid (h-FF).
